# Chronic pain in Asia: we don't have to endure

**DOI:** 10.1016/j.lanwpc.2023.100782

**Published:** 2023-04-28

**Authors:** The Lancet Regional Health – Western Pacific

The Chronic Pain Series, published by *The Lancet* on May 27, 2021, highlighted the burdens and progress of chronic pain management. Globally, one in five people have chronic pain, but some endure more than others. Women, older populations, and people of a lower socioeconomic status are more likely to report pain. Due to the ageing population and the high burden of chronic diseases, chronic pain is becoming a serious health-care challenge in the Asia–Pacific region. Depending on the use of different methods to measure pain, the prevalence of chronic pain in Asian countries varies from 7.1% to 90.8% among adults. However, awareness and management of pain have long been neglected.

Chronic pain is defined as any pain lasting longer than 12 weeks. Unlike acute pain, which functions as a biological warning system, chronic pain is a complex health condition dynamically affected by biological, psychological, and social factors. It includes many conditions such as cancer pain, neuropathic pain, and musculoskeletal pain. Each of them carries heterogeneous symptoms that might require different approaches for diagnosis and treatment. Untreated chronic pain adversely affects patients’ physical and mental health, impairs quality of life, and reduces work productivity.

Cultural beliefs shape people's understanding, experience, and responses to pain, including health-seeking behaviour. In many Asian cultures, patients might not seek medical help because chronic pain is regarded as an expected part of life and a normal ageing process that should be endured. A recent survey study on chronic pain from China showed that 24.06% of respondents did not seek medical help, and 36.79% of patients never received any treatment because they believed chronic pain is not harmful.

Pain is a subjective phenomenon, and pain assessment and diagnosis rely largely on patients’ self-report. The complicated aetiology for chronic pain and limited medical resources and staff specialising in pain management contribute to the underdiagnosis of chronic pain in clinical settings. The standard pain assessment process is the initial step for accurate diagnoses and effective treatment, but it is usually inadequately used. In a multinational survey (Current practices of cancer and chronic non-cancer pain management: a pan-Asian study [ACHEON]), 49.5% of patients with chronic cancer pain mentioned that they did not receive pain scale assessment in diagnosis. There are also discrepancies between patients and physicians in pain rating, and patient pain is often underestimated in the clinical setting. In another ACHEON study, authors found that patients with chronic non-cancer pain are more willing to report their pain when it becomes more severe and when they have sufficient time to talk with physicians. This situation can be improved by regular pain measurement and, more importantly, changes in physician training on pain management by shifting the focus towards relieving pain and not just treating diseases causing pain only.

Chronic pain is a complex phenomenon that needs to be managed using the biopsychosocial model, requiring the involvement of multimodal and multidisciplinary approaches, including self-management tools such as exercise and lifestyle changes, opioid and non-opioid pharmacological therapies, psychological therapies, and integrative treatments. Globally, chronic pain is commonly not optimally managed. The *Lancet* Series on low back pain indicated the overuse of low-value care and the underuse of high-value care for low back pain management. Although the recommended first-line treatment for low back pain is non-pharmacological therapies, this suggestion is usually not followed. For low-income and middle-income countries, quite often the local health-care system has no capacity to deliver non-pharmacological care. As an alternative, complementary and alternative medicine are widely used for pain management in Asia; however, high-quality evidence for the efficacy is still limited.

Although not recommended for chronic pain management, opioids are an effective medicine for relieving cancer-related pain and are included on the WHO Essential Medicines list. The use of opioids in Asia in 2019 was almost four times lower than the global opioid analgaesic consumption level, mainly due to the stringent regulations and stigma associated with opioid analgaesics. However, the expansion in opioid choice alone does not improve opioid prescription. To provide more options to relieve pain for patients with cancer, the strong opioids hydromorphone and oxycodone were introduced in Taiwan in 2014. Although the newly introduced strong opioid use rate increased during the study period, the weak opioid use rate and the use of guideline-recommended strong opioids morphine and transdermal fentanyl, which existed before 2014, decreased, thereby decreasing the total opioid use in Taiwan. In the linked commentary, Masahiko Sumitani from the University of Tokyo Hospital suggests that to improve the availability of opioid analgaesics, addressing supply problems alone is insufficient; improved awareness and knowledge about pain management among patients and health-care professionals are also needed in east Asian countries.

Cultural beliefs, physician knowledge, and poor access to analgaesics are considered major barriers contributing to suboptimal chronic pain management in Asia. Improving the wellbeing of patients with chronic pain relies on raising awareness of chronic pain management among the public and health professionals and adapting biopsychosocial models for pain assessment and treatment. Our current understanding of pain aetiology and management is mainly based on research from Europe and North America. There are differences in patient attitudes, professional training, and health-care system preparedness towards chronic pain between countries, which influence pain management and treatment outcomes. More local research on chronic pain is needed to give it a voice in the Asia–Pacific region. As a regional health-care knowledge platform, *The Lancet Regional Health – Western Pacific* welcomes all research and opinion pieces on chronic pain from the region.

